# *β*Lact-Pred: A Predictor Developed for Identification of Beta-Lactamases Using Statistical Moments and PseAAC via 5-Step Rule

**DOI:** 10.1155/2021/8974265

**Published:** 2021-12-17

**Authors:** Muhammad Adeel Ashraf, Yaser Daanial Khan, Bilal Shoaib, Muhammad Adnan Khan, Faheem Khan, T. Whangbo

**Affiliations:** ^1^Department of Computer Science, University of Management and Technology, Lahore 54770, Pakistan; ^2^Department of Computer Science, Minhaj University Lahore, Lahore 54770, Pakistan; ^3^Centre of Research and Innovation in Marytime Affairs (CRIMA), Lahore 54770, Pakistan; ^4^Pattern Recognition and Machine Learning Lab, Department of Software, Gachon University, Seongnam 13120, Republic of Korea; ^5^Department of Computer Engineering, Gachon University, Seongnam 13120, Republic of Korea

## Abstract

Beta-lactamase (*β*-lactamase) produced by different bacteria confers resistance against *β*-lactam-containing drugs. The gene encoding *β*-lactamase is plasmid-borne and can easily be transferred from one bacterium to another during conjugation. By such transformations, the recipient also acquires resistance against the drugs of the *β*-lactam family. *β*-Lactam antibiotics play a vital significance in clinical treatment of disastrous diseases like soft tissue infections, gonorrhoea, skin infections, urinary tract infections, and bronchitis. Herein, we report a prediction classifier named as *β*Lact-Pred for the identification of *β*-lactamase proteins. The computational model uses the primary amino acid sequence structure as its input. Various metrics are derived from the primary structure to form a feature vector. Experimentally determined data of positive and negative beta-lactamases are collected and transformed into feature vectors. An operating algorithm based on the artificial neural network is used by integrating the position relative features and sequence statistical moments in PseAAC for training the neural networks. The results for the proposed computational model were validated by employing numerous types of approach, i.e., self-consistency testing, jackknife testing, cross-validation, and independent testing. The overall accuracy of the predictor for self-consistency, jackknife testing, cross-validation, and independent testing presents 99.76%, 96.07%, 94.20%, and 91.65%, respectively, for the proposed model. Stupendous experimental results demonstrated that the proposed predictor “*β*Lact-Pred” has surpassed results from the existing methods.

## 1. Introduction

The advent of penicillin was a great revolution of the last century in the medical history of mankind. It was a very effective treatment for many incurable diseases of that time and led to the discovery of more effective remedies for other fatal diseases. After this substantial discovery, a large number of antibiotics were discovered to kill disease-causing bacteria. As the application of such advanced drugs increased, bacteria also acquired resistance to these antibiotics by producing enzymes capable of breaking down these antibiotics [1]. One example of such an antibiotic-resistant enzyme is beta-lactamase which hydrolyzes the beta-lactam ring found in antibiotics, thus destroying its structure. Consequently, effective antibiotic medications are formed by administering the *β*-lactam antibiotic drug along with a beta-lactamase inhibitor to cure a bacterial infection [2]. In this perspective, *β*-lactam antibiotics and *β*-lactamases are of great consideration in clinical set up for the treatment of skin infections, respiratory tract infections, eye infections, gonorrhoea, soft tissue infections, bronchitis, meningitis, urinary tract infections, pneumonia, and others. A lot of work has been done to understand the structure and the action mechanism of these enzymes in order to elucidate the acquired immunity of microbes against different drugs [3]. *β*-Lactamase enzymes are produced from bacteria such as cephamycins, penicillins, cephalosporins, and carbapenems [4, 5]. Its action mechanism works by breaking down the beta-lactam ring present in all broad-spectrum antibiotics through hydrolysis, thus deactivating the antibacterial nature of the drug. These antibiotics are used to treat a vast spectrum of Gram-negative and Gram-positive bacterial infections though *β*-lactamases are produced only from Gram-negative and anaerobic bacteria [5].

**Figure 1:**
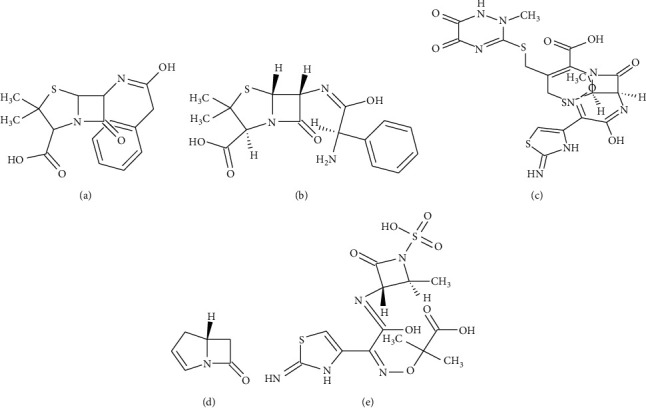
Chemical structures of the *β*-lactam antibiotics. (a) Penicillin. (b) Ampicillin. (c) Cephalosporin. (d) Carbapenem. (e) Monobactam.

Figure 1 depicts the chemical structure of different *β*-lactam antibiotics. The ring of *β*-lactam is can be seen as a quad-edge shape for each antibiotic [6]. Three classes of these enzymes, i.e., A, C, and D hydrolyze the substrate by making an acyl-enzyme with the active involvement of serine residue. While class B enzyme uses Zn+ for carrying out its normal function [6].

The initial work of Yildirim et al. studied a ligand based on network model to cluster proteins. A network was created, and the target protein network was connected to their node if there was at least one ligand common. However, the study demonstrated results pertaining to only common networks and not for different compounds [7]. Keiser et al. used ligand-based chemical resemblance and formulated subsets of ongoing classes [8]. Cheng et al. used a bipartite network to represent the target node and the protein compound on the basis of similarity sharing protein and ligand [9, 10]. In 2009, Bailey et al. worked on uses of MEME-MAST to extract motifs on the amino acid sequence in *β*-lactamase [11]. Both works do not concern chemical applications. But since the fuzzy techniques are “data independent,” they can also be exploited for the problem under study by the authors [12, 13]. Recently, a predictor named Blapred has been proposed for the classification and identification *β*-lactamases with its respective classes, i.e., A, B, C, or D by using a three-tier identification computation model via Chou's PseAAC [14].

In the past, chemists and biologists used traditional methods to identify and differentiate of a protein in the laboratory with the utilization of costly equipment which is time-consuming, operator-dependent, costly, and laborious. Besides this, the predictors previously available to classify and identify *β*-lactamase do not have higher accuracy [14]. There is a need to construct a computational model for the differentiation and classification of *β*-lactamase enzymes from non-*β*-lactamase enzymes. The objective of the research is to develop a computational model *β*Lact-Pred by collecting a benchmark dataset, extracting the features and then training the model via Chou's PseAAC [15]. For the purpose of identification and differentiation of proposed model, Chou's five steps are employed which entails [16, 17] (i) construction or selection of an effective benchmark dataset for training and testing the sequence-based statistical predictor, (ii) using mathematical expression, finding a correlation in the dataset, which is called feature extraction; (iii) implementing an algorithm for learning and prediction; (iv) performing numerous kind of persuasive verification and validation testing to factually assess the projected precision of the predictor. This tells that how much our method is effective and trustworthy; (v) developing of a comprehensible and foolproof webserver that will be user-friendly, to ensure its receptiveness and accessibility to the public.

## 2. Methods and Materials

**Figure 2:**
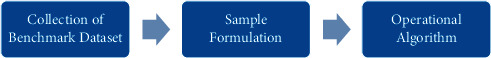
Graphical illustration of the computational model using the Chou's first three stages.

Consecutively, to develop a vigorous computational model, it is prerequisite to acknowledge an accurate and explicit scale dataset for the sake of training and testing the model. An inoperative dataset may lead the computational model to produce capricious results with untrustworthy validation and unyielding verification testing. It is of uttermost suggestive that the gathered dataset is an accurate, pertinent, nonredundant, related, and comprehensive. Protein's sequence dataset is collected to construct the *β*Lact-Pred computational model. Important and relevant statistical feature vectors are extracted in the form of numerical from the essential protein structure/primary sequences. The computational model is trained on these extracted features using the neural network to accomplish the convergence. Here, Chou's first 3-steps will remain tended, as illustrated in Figure 2.

### 2.1. Collection of Benchmark Data Set

A database which is publicly available and well-known named Uniport is the major fount to collect the protein sequences of beta-lactamase and non-beta-lactamase. To acquire the concerning positive sequences “beta-lactamase” named keyword was used. An accurate and meticulously process is used to collect dataset in which ambiguous, dubious, and uncertain sequences are excluded, by probability or similarity. Furthermore, for the purpose of accurate and valid results, complete sequences which should not be annotated with fragment-like words are selected. These sequences are annotated with different class names, e.g., class A, B, C, or D. To exclude the redundant and homology-biased sequences, CD-HIT [17] is used with ≥60% resemblance. In consequence, a great quality and an excellent data set is collected which includes the most up-to-date beta-lactamase protein sequences.

After applying CD-HIT, 2172 beta-lactamase sequences were derived. By following the same procedure, 3463 non-beta-lactamase were derived from the same database named UniProt. By considering the Chou's rule [18], any protein sequence can be illustrated as

(1) $$\begin{array}{c} K_{\rho }\left(\beta \right)=M_{0}M_{1}\ldots M_{\left(n-1\right)}M_{n}. \end{array}$$

Considering all, a minimized dataset was obtained by the following equation:

(2) $$\begin{array}{c} T=T^{+}\cup T^{-}. \end{array}$$

Here, *T*^+^ contains 2172 positive beta-lactamase sequences, *T*^−^ contains 3463 negative beta-lactamase sequences, and ∪ shows the “union of two set.” A total of 5635 (2172 + 3463 = 5635) sequences comprised dataset.

### 2.2. Sample Formulation

A specific sequence is constructed by using the amino acids polypeptide chain. These sequences contain biophysical characteristics of proteins. Minor absence or presence of amino acids could not control the characteristics of protein. Behavior of protein is contrived by many constituents, e.g., positioning of amino acids residues and their composition. By observing data and the behavior of different models, it is noted that minor change in comparative composition or ordering of amino acids residue change the characteristics of protein by great extent. Due to all these facts, feature vectors are extricating from primary or core building/blocks of protein by using the computational model which contains both of amino acids relative positions and protein constituents. An extended technique from the technique [18, 19] is used to extract features for *β*Lact-Pred.

#### 2.2.1. Statistical Moment Calculation

Quantitative measures to describe the collection of data are known as statistical moments. Different statistical moments order renders nonidentical data properties. Some statistical moments are helpful in evaluation of the data size, some demonstrate data eccentricity, and some are related to the alignment of proteins. These moments formed by some mathematicians and statisticians contain certain polynomials and distribution functions. *β*Lact-Pred explained by using the moments which include Central, Raw, and Hahn moments. Raw moments, most fundamental moments, contain different properties of a distribution, e.g., mean, variance, and asymmetry. Raw moments do not represent the location, rotation, and scale invariants. To calculate location, rotation, and scale invariants, central moments are calculated deliberately. Central moments again did not calculate the scale and location variants. To calculate scale and location variant properties, another well-liked set of moments named Hahn moment is computed. Hahn moment obtained by using Hahn polynomials exhibits scale and location variants. Major keys to choose these moments are to inspect the composition and composition of residues as they are important factors as per initial discussion. Calculated values yielded from the all above techniques describe in data in their distinctive way. Furthermore, variance is described in terms of moments by using numerical values for capricious datasets [20].

To make protein synthesis, solely 20 amino acids are useable. To compute the moments, distinctive integer index is allocated to each and every amino acids residue. If the allocated index is unique, consistent, and integral, then it barely makes any distinction that what a particular esteem is substituted. Initially, a mapping conversion tool is discovered to convert 1-D (one-dimensional) essential structure into a 2-D (two-dimensional) illustration by equation.

Let *S* be a sequence of the proteins. The format of *S* is given as follows:

(3) $$\begin{array}{c} S=\left\{\beta_{1},\beta_{2},\beta_{3},\ldots ,\beta_{m-1},\beta_{m}\right\}. \end{array}$$

In above, *m* is surplus in primary protein

(4) $$\begin{array}{c} Z=\;\left\lceil \sqrt{m}\right\rceil , \end{array}$$

where *Z* represents the features of *S*′ matrix in the following equation.

All amino acids *S* that are computed given by *m∗m*

(5) $$\begin{array}{c} S^{\prime }=\left[\begin{array}{cccc} K_{11} & K_{12} & \cdots & K_{1n}\\ K_{21} & K_{22} & \cdots & K_{2m}\\ \vdots & \vdots & \ddots & \vdots \\ K_{m1} & K_{m2} & \ldots & K_{mm} \end{array}\right]. \end{array}$$

The 2-D matrix *S*′ refers to matrix *S*. It can be converted by using mapping function as *ν*.

(6) $$\begin{array}{c} \nu \left(\beta_{x}\right)=\alpha_{pq}, \end{array}$$

where *p* and *q* signify the index of *K* in *S*′.

Moments can be computed till 3-degree by using two-dimensional *S*′, and consequent equation is utilized for computing raw moments.

(7) $$\begin{array}{c} Z_{mm}=\sum_{x=1}^{l}\sum_{y=1}^{l}x^{m^{y^{n}a_{xy}}}, \end{array}$$

where *m*+*n*] indicates the order of moments, *l* describes the aspects of matrix, which should be the same, i.e., *Z*. Moments till 3-degree are computed as *Z*_00_, *Z*_01_, *Z*_02_, *Z*_10_, *Z*_11_, *Z*_12_, *Z*_20_, *Z*_21_, and *Z*_22_.

Data center is like center of gravity. Distribution of data is fair along with the data's central point w.r.t the average weight of data. It computes the following raw moments and known as an argument $\left(\bar{v},\bar{w}\right)$, where

(8) $$\begin{array}{c} \bar{v}=\frac{Z_{10}}{Z_{00}},\\ \bar{w}=\frac{Z_{01}}{Z_{00}}. \end{array}$$

Central moments are calculated by point where the centroid is acting. The following equation is employed to compute the central moments such as

(9) $$\begin{array}{c} B_{st}=\sum_{k=1}^{m}\sum_{l=1}^{m}{\left(k-\bar{v}\right)}^{s}{\left(l-\bar{w}\right)}^{1}a_{kl}. \end{array}$$

For Hahn moments calculation, 1-D analysis *S* was transferred to a square matrix analysis *S*′. The Hahn polynomials in *n* order can be employed as

(10) $$\begin{array}{c} \omega_{m}^{a,b}\left(p,M\right)={\left(M+b-1\right)}_{m}{\left(M-1\right)}_{m}\times \sum_{l=0}^{m}{\left(-1\right)}^{l}\frac{{\left(-m\right)}_{l}{\left(-p\right)}_{l}{\left(2M+a+b-m-1\right)}_{l}}{{\left(M+b-1\right)}_{l}{\left(M-1\right)}_{l}}\frac{1}{l!}. \end{array}$$

The above polynomial uses Pochhammer mark as

(11) $$\begin{array}{c} {\left(b\right)}_{l}=b,\left(b+1\right)\ldots \left(b+1-1\right). \end{array}$$

Simple form of the above can be represented by using a delta operator:

(12) $$\begin{array}{c} {\left(b\right)}_{l}=\frac{\Delta \left(b+l\right)}{\Delta \left(b\right)}. \end{array}$$

Hahn moments are calculated by weighing function and square rule such as

(13) $$\begin{array}{c} {\tilde{\beta }}_{n}^{c,d}\left(q,N\right)=\beta_{n}^{c,d}\left(q,N\right)\sqrt{\frac{ο\left(q\right)}{c_{n}^{2}}}\;n=0,1,\ldots N-1, \end{array}$$

whereas

(14) $$\begin{array}{c} ο\left(q\right)=\frac{\phi \left(c+q+d\right)\phi \left(d+q+1\right){\left(c+d+q+1\right)}_{N}}{\left(c+d+2q+1\right)n!\left(N-q-1\right)!}. \end{array}$$

The logical data for 2-dimentional discrete data is calculated by using the following equation:

(15) $$\begin{array}{c} G_{ef}=\sum_{c=0}^{N-1}\sum_{d=0}^{N-1}\alpha_{c\;d}{\tilde{J}}_{t}^{g,h}\left(c,N\right){\tilde{J}}_{s}^{u,v}\left(b,N\right),\;n=0,1,\ldots N-1. \end{array}$$

In order, Han and Central moments can be calculated up to 3.

#### 2.2.2. Generation of Position Relative Index Matrix

Information regarding the composition/arrangements is the foundation of any computational model that is used to predict protein functions. Physical properties of the proteins can be determined by assuming a key function for the area of amino acid. Relative positioning of amino acid in polypeptide chain is very important as position relative index matrix (PRIM) divulges information about the relative position of amino acids in polypeptide chain. Position relative index matrix (PRIM) excerpts the amino acid's location information in polypeptide chain [20]. A matrix of 20 × 20 dimensions related to PRIM matrix is given as follows:

(16) $$\begin{array}{c} Z_{\mathrm{PRIM}}=\left[\begin{array}{cccccc} Q_{1⟶1} & Q_{1⟶2} & Q_{1⟶3} & Q_{1⟶b} & \cdots & Q_{1⟶20}\\ Q_{2⟶1} & Q_{2⟶2} & Q_{2⟶3} & Q_{2⟶b} & \cdots & Q_{2⟶20}\\ \vdots & \vdots & \vdots & \vdots & \cdots & \vdots \\ Q_{d⟶1} & Q_{d⟶2} & Q_{d⟶3} & Q_{d⟶b} & \cdots & Q_{d⟶20}\\ \vdots & \vdots & \vdots & \vdots & \cdots & \vdots \\ Q_{U⟶1} & Q_{U⟶2} & Q_{U⟶3} & Q_{U⟶b} & \cdots & Q_{U⟶20} \end{array}\right]. \end{array}$$

An element of matrix such as *Q*_*d*⟶*b*_ contains the aggregate of *b*^th^ residue in contradiction of the first index of *d*^th^ residue. It makes 400 coefficients which show a large number. Dimensions of PRIM matrix are curtailed by computing the three moments, i.e., raw, central, and Hahn.

#### 2.2.3. Generation of Reverse Position Relative Index Matrix (RPRIM)

Reverse position relative index matrix (RPRIM) is used to extract hidden features from protein sequences which have the ambiguity of homologous sequences. RPRIM has a 20 × 20 dimension matrix containing 400 coefficients same as in the PRIM, but it is used in a reverse order of the PRIM [20].

(17) $$\begin{array}{c} Q_{\mathrm{RPRIM}}=\left[\begin{array}{cccccc} R_{1⟶1} & R_{1⟶2} & \cdots & R_{1⟶k} & \cdots & R_{1⟶20}\\ R_{2⟶1} & R_{2⟶2} & \cdots & R_{2⟶k} & \cdots & R_{2⟶20}\\ \vdots & \vdots & \cdots & \vdots & \cdots & \vdots \\ R_{t⟶1} & R_{t⟶2} & \cdots & R_{t⟶k} & \cdots & R_{t⟶20}\\ \vdots & \vdots & \cdots & \vdots & \cdots & \vdots \\ R_{z⟶1} & R_{z⟶2} & \cdots & R_{z⟶k} & \cdots & R_{z⟶20} \end{array}\right]. \end{array}$$

Like PRIM, the dimension of the RPRIM matrix is also curtailed by computing the three moments, i.e., raw, central, and Hahn.

#### 2.2.4. Frequency Matrix

Frequency matrix is a technique used to determine the structure and how frequently proteins are occurring. This plays a significant role in sequencing of proteins. PRIM holds the series information of amino acids, while frequency matrix does not hold that series information [20]. The following expression is used to compute the frequency of the matrix as

(18) $$\begin{array}{c} \xi =\left\{\tau_{1},\tau_{2},\tau_{3},\tau_{4}\;,\ldots ,\tau_{20}\right\}. \end{array}$$

Here, *τ*_*i*_ denotes the frequency of *i*^th^ essential amino acid.

#### 2.2.5. Generation of Accumulative Absolute Position Index Vector

Frequency matrix contains the protein formation related information and the total occurrence of protein information. Frequency matrix did not contain the information related to the occurrence of amino acid residues in a polypeptide chain. Accumulative absolute position incidence vector (AAPIV) is used to compute the information related to the position of amino acid residue in the polypeptide chain. AAPIV contains position relevant information in a vector form. A vector with 20 elements in which each component encompasses a numerical ordered value to represent the amino acid position relevant information from the residue [20]. Native sequence shows the specific residue occurrence in a protein structure which is given as follows:

(19) $$\begin{array}{c} \upsilon_{\mu^{1}}^{k}\ldots \upsilon_{\mu^{2}}^{k}\ldots \upsilon_{\mu^{3}}^{k}\ldots \upsilon_{\mu^{n}}^{k}. \end{array}$$

It represents *υ*^*k*^ residue which is placed at a position of *μ*^1^,*μ*^2^,*μ*^3^,…*μ*^*n*^

Let accumulative absolute position index vector represented as

(20) $$\begin{array}{c} T=\left\{\nu_{1},\nu_{2},\nu_{3},\nu_{4},\ldots ,\nu_{20}\right\}. \end{array}$$

Hence, *i*^th^ element of the accumulative absolute position index vector is computed by

(21) $$\begin{array}{c} v_{i}=\sum_{u=1}^{n}su. \end{array}$$

#### 2.2.6. Generation of Reverse Accumulative Absolute Position Index Vector

As per earlier discussion, detecting ambiguous patterns using feature extraction is an efficient technique. RAAPIV did the same task as AAPIV performs, but it finds the patterns in a reverse order [20]. It also contains 20 elements which can be represented as follows:

(22) $$\begin{array}{c} \delta =\left\{{ο}_{1},{ο}_{2},{ο}_{3},{ο}_{4},{ο}_{5},\ldots ,{ο}_{20}\right\}. \end{array}$$

Reversed sequence in RAAPIV is shown as

(23) $$\begin{array}{c} \omega_{m1}^{k}\ldots \omega_{m2}^{k}\ldots \omega_{m3}^{k}\ldots \omega_{mn}^{k}. \end{array}$$

The amino acid residue _*ω*_^*k*^ that occurs in the reverse order sequence and the term *m*_1_, *m*_2_, *m*_3_,…, *m*_*n*_ represents their ordered position. The significance of any residue is calculated as

(24) $$\begin{array}{c} \ell_{i}=\sum_{m=1}^{n}t_{m}. \end{array}$$

All of these abovementioned features have specific biological significance. These methods help in extracting position and composition relative features from the amino acid sequence which is a very pivotal aspect while dealing with proteins. Each amino acid, in its surrounding, plays a role in describing the physiochemical characteristics of that molecule; thus, these features help in extracting such information. For example, the frequency of amino acids in molecule, position relative occurrence of amino acids, composition of a specific peptide, and absolute positioning of residues.

## 3. Operational Algorithm via Neural Network

**Figure 3:**
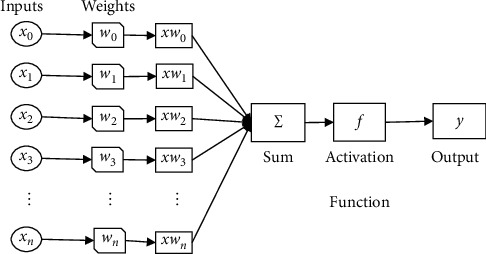
Graphical representation of the artificial neural network for *β*Lact-Pred.

Artificial neural network is one of the most significant tools for tackling the issue examined in this paper, it mimics preparing data as depicted in Figure 3. Neural network clarifies the fundamental shape of every residue within a protein. To train the model, composition of positive and negative feature vectors which are extracted in above section are used. These feature vectors depict the two-dimensional structure of protein by using central, raw, and Hahn moments. Here, in this study, the neural network was considered as neural network which is represented by directed graph similar to the biological neuron system in brain. Back propagation ANN was used instead of SVM because of many reasons that ANN performs better than SVM. First of all, ANN is a parametric model, while SVM is not. As in ANN, there can be many hidden layers depending on features and parameters [20]. In SVM, we have support vectors that are acquired by training data. In some cases, support vectors can have many support vectors with weight of each vector. ANN can also have one or many outputs, while SVM can have only one output. In case of a *n*-ary classifier, ANN can be trained in one step, while SVM needs to train *n* support vectors one by one that is time-consuming [20].

ANN is fast and flexible. ANN can be reached at global optimal point, and we do not face any issue regarding choosing the number of parameters, but in case of SVM, we need to select hyperparameters. Less amount of memory is required to store ANN, but SVM requires much memory because it needs to store support vectors as well. Results in ANN are more readable and interpretable [21, 22].

## 4. Formulation of Results and Discussion

### 4.1. Estimated Accuracy Metrics

The unbiased assessment of newly constructed computational model is the most key aspect that aids to estimate the accomplishment of that computational model [22, 23]. Conversely, for such kind of an unbiased assessment, two important aspects one must keep in mind that (i) the choice of metrics accuracy and (ii) the test method deployed for the validation of the computational model. Here, first classify the measurements for the unbiased assessment and then use the numerous validation and verification techniques.

### 4.2. Mathematical Formulation of Metrics

It is obvious that, for any machine learning problem, some collective and important metrics are used for formulation of the metrics, which are (1) Acc (accuracy) is the percentage of correctly classified samples from total input dataset; (2) MCC (Matthews correlation coefficient) is used in case of binary classification, and it is also considered as balanced measure even in multiple classes of different sizes; (3) *S*_*n*_ (sensitivity) is the percentage of true positive or those samples that are correctly classified as positive, and it is also called true positive recognition rate. (4) *S*_*p*_ (specificity) is the percentage of true negative or those samples that are correctly classified as negative, and it is also called true negative recognition rate.

Predominantly, these four metrics were introduced in 2001, and an accurate set of four measures was obtained in [24] for all of these measures.

(25) $$\begin{array}{c} \left\{\begin{array}{cc} \mathrm{Sn}=1-\frac{{Ŋ}_{-}^{+}}{{Ŋ}^{+}} & 0\leq \mathrm{Sn}\leq 1\\ \mathrm{Sp}=1-\frac{{Ŋ}_{+}^{-}}{{Ŋ}^{-}} & 0\leq \mathrm{Sp}\leq 1\\ \mathrm{Acc}=1-\frac{{Ŋ}_{-}^{+}+{Ŋ}_{+}^{-}}{{Ŋ}^{+}+{Ŋ}^{-}} & 0\leq \mathrm{Acc}\leq 1\\ \mathrm{Mcc}=\frac{1-\left(\left({Ŋ}_{-}^{+}/{Ŋ}^{+}\right)+\left({Ŋ}_{+}^{-}/{Ŋ}^{-}\right)\right)}{\sqrt{\left(1+\left({Ŋ}_{+}^{-}-{Ŋ}_{-}^{+}/{Ŋ}^{+}\right)\right)\left(1+\left({Ŋ}_{-}^{+}-{Ŋ}_{+}^{-}/{Ŋ}^{-}\right)\right)}} & -1\leq \mathrm{Mcc}\leq 1 \end{array}\right). \end{array}$$

Here Ŋ^−^ signifies non-*β*-lactamases data, predicted as non-*β*-lactamases correctly by *β*Lact-Pred. Ŋ_+_^−^ signifies the non-*β*-lactamases aggregate number which are anticipated inaccurately as *β*-lactamases by *β*Lact-Pred. Additionally, Ŋ^+^ is the *β*-lactamases aggregate number which are predicted correctly as *β*-lactamases by *β*Lact-Pred, and Ŋ_−_^+^ is the *β*-lactamases aggregate number which are identified inaccurately as non-*β*-lactamase by *β*Lact-Pred. Accordingly, equation (25) provides the information regarding Sn, Sp, Acc, and consistency more relaxed to recognize and innate, especially when we discourse about MCC [25, 26].

These accuracy metrics have been used/identified by a numerous researchers [27, 28], but merely for binary class data labelled. Multiclass data labelled identification is a utterly diverse problem, which has been supplementary prominent in computational biology [29] and biomedicine [30]. Consequently, it entails a diverse kind of accuracy metrics for formulation [29].

### 4.3. Self-Consistency Testing

The self-consistency testing is a term referred as the ultimate test for the validation of efficiency and efficacy of the prediction model using the test cases by training the data set. The reason behind the implementation of self-consistency is that the obtained results are individual and the actual true positive rate of the benchmark dataset is also known. Self-consistency results are revealed in Table 1; it can be observed that the *β*Lact-Pred has the 99.76% Acc, 99.76% Sp, 99.76% Sn, 0.99 MCC, and 0.99 AUC.

### 4.4. Validation of Model via Leave-One-Out

Validation is a significant step that comes toward the end of the process. Its motivation is to discover that how much the model is proficient. A few validation techniques are utilized to validate the model. To validate the model, data are portioned into two parts; (1) training set and (2) testing set. The model is trained on training data, and then its performance is measured on testing data. As the validation techniques select the data haphazardly for predicting the model, there is not well-defined technique that expresses how to partition the data from the given dataset. Generally, the predictive model can be tested using numerous types of testing, i.e., k-folds (subsampling), independent testing, and leave-one-out (jackknife) [27, 30]. Jackknife testing is amongst the most frequently used validation techniques. Jackknife works by overlooking each observation from the data and set up the model on residual data. At the end, average is calculated of all calculations and the output is unique. Issues like sampling or sub-sampling are alleviated.

Jackknife is used to quantify the quality of the predictor, and it is likewise generally utilized in these sorts of problems. It is an iterative technique that computes the accuracy of the model for all variations of the sample of size *n* − 1. The jackknifing technique trains the predictor on left-out data and estimates overall accuracy by meticulously leaving out every observation from a dataset. It is more efficient as it overwhelms the issues that are triggered by data independency and subsampling [31]. Results of jackknife validation testing is 96.07% which is higher than the BlaPred [12] and are revealed in Table 2.

### 4.5. K-Fold Cross-Validation Testing

Cross-validation is a method to thrive an expectancy for the proposed model as an exemplary method in the absence of validation set. Cross validation tests the model on given training dataset and prevents underfitting and overfitting. In *k*-fold cross validation, the dataset is portioned into *k* sets and *k* is picked at start, and afterward, it is kept constant. Generally, *k* is kept 5 or 10; however, in the proposed method, *k* is set to 10. The model is tested *k* times and, in each iteration, 9 sets (*k*-1) are used for training set and the one set (*k* set) is treated as testing set. Subsequent to performing *k* iterations, the accuracy of model is computed by the sum of each iteration and then divided by *k*. This average accuracy is considered as a result of cross validation. The overall 10-fold validation was repeated 20 times, so that the credibility of results is increased, as illustrated in Table 3.

### 4.6. Independent Dataset Testing

To evaluate the precision of *β*Lact-Pred, independent testing was performed, in which the training/testing split method was used for validating the model. Out of 2172 positive and 3463 negative samples, three different train/test split ratios were used which were 90/10, 80/20, and 70/30. After sufficient training, the left-out samples were used for testing, and subsequent evaluation of the accuracy of the proposed prediction technique was performed. Based on the ability and inability of the model to recognize the test samples accurately, all the described metrics in equation (25) were computed, which are mentioned in Table 4.

### 4.7. Comparative Analysis

*β*Lact-Pred uses a composition and position variant feature extraction method for classification besides neural network. The other existing prediction models discussed in text use type-1 PseAAC, type-2 PseAAC, and classic PseAAC for feature extraction combined with SVM (support vector machine). Both the techniques (type I and type II) and classic are based on the PseAAC model, presented in [32]. The method of feature extraction for such kind of problems has extreme significance. The proficiency to uncover deeply obscure patterns within a specified set of data is highly anticipated for a feature extraction algorithm. The capability of a model to translate deeply obscure patterns in the primary structure into coefficients is dependent on a variable *λ*. The value of *λ* not only determines the size of the feature vector but also plays a significant role in sieving out the correlation among residues within a peptide chain. The factors produced by *β*Lact-Pred are not reliant on such a variables. The vector size of the feature is adjusted and carefully calculates all possible interactions between all possible residues in the peptide chain in the form of succinct. *β*Lact-Pred used both assorted sequences of *β*-lactamase and non-*β*-lactamase which is subsequently used as a dataset for the purpose of training and testing. As illustrated in Table 1, *β*Lact-Pred reveals a greater sensitivity, specificity, accuracy, and MCC for prediction of *β*-lactamases and non-*β-*lactamases than the other previous predictors. Experiments prove that it is a highly efficient technique as compared to previous ones. Rigorous validation in diverse scenarios elucidates that the method is less noisy and more effective for the prediction of beta-lactamases. Subsequently, it is also established that the presented methodology provides higher throughput and accuracy than the previous predictors. To quantitatively evaluate and compare the *β*Lact-Pred, an independent dataset of 75 *β*-lactamases, previously reported by [12], was used in (Table 5).

In addition to this, the results of *β*Lact-Pred were also compared with CNN-BLPred [33], which performs the functional and molecular classification of *β*-lactamases by employing a deep learning method/technique called the convolutional neural network (CNN). The study performs classification of *β*-lactamases at molecular and functional level; however, for comparison with *β*Lact-Pred, only molecular classification (level 1) results were considered. Comparative analysis is provided in Table 6.

Furthermore, *β*Lact-Pred applies numerous types of approach and uses composition and positioning features of sequences of protein to accomplish the identification of *β*-lactamases. In first, it uses PseAAC, and then it calculates the statistical moments, AAPIV, RAAPIV, PRIM, and RPIRM using the relative positioning features of protein; thus, *β*Lact-Pred outperforms its counterparts.

## 5. Web Server

Final step of Chou is the enlargement of user-friendly and publicly accessible webserver for the comfort of chemists and biologists as an enlightened in [34, 35]. Publicly accessible and user-friendly webserver development and establishment signifies the direction of the future in order to develop prediction methodologies [34, 35]. For this purpose, various computational analysis and research findings have been reported. Therefore, useful and practical webserver has significantly enhanced the overall impacts of computational biology on medical sciences directing medicinal chemistry into an unsurpassed revolution [12]. In this view, the webserver shall be established for *β*Lact-Pred as described in the paper.

## 6. Conclusion

Multidrug-resistant strains of bacteria have posed a great threat to human health nowadays. Bacteria have cleverly and speedily acquired resistance against most of the antibiotics of the time and are creating hurdles in an effective cure for diseases. It is believed that, within few years, all prevailing antibiotics would lose their efficacy against these multidrug-resistant bugs. *β*-Lactamase is one of the safeguards produced by bacteria which protects it from the adverse action of *β*-lactam antibiotics. Various data preprocessing techniques are used to calculate the feature vector including raw, Hahn, and central Moments and position and composition variant features. For this purpose, an artificial neural network is used for training and predicting the sequences. The results for the proposed computational model was validated by employing numerous types of approaches, i.e., self-consistency testing, jackknife testing, cross-validation, and independent testing. The overall accuracy of the predictor for self-consistency testing, jackknife testing, cross-validation, and independent testing by using paradigm metrics presents 99.76%, 96.07%, 94.20%, and 91.65%, respectively, for the proposed model. Stupendous experimental results demonstrated that the proposed predictor “*β*Lact-Pred” has surpassed results from the existing methods.

## Figures and Tables

**Table 1:** Performance analysis of self-consistency for *β*Lact-Pred.

Predictor/identifier	Precision metrics				
	Acc (%)	Sp (%)	Sn (%)	MCC	AUC
*β*Lact-Pred	99.76	99.76	99.76	0.99	0.99

**Table 2:** Jackknife testing results for *β*Lact-Pred.

Predictor/identifier	Precision metrics				
	Acc (%)	Sp (%)	Sn (%)	MCC	AUC
*β*Lact-Pred	96.07	97.39	96.96	0.92	0.93
BlaPred [12]	93.57	94.00	89.24	0.70	—

**Table 3:** Performance analysis of 10-fold cross-validation results (20 iterations) for *β*Lact-Pred.

10-fold (iterations)	Precision metrics				
	Acc (%)	Sp (%)	Sn (%)	MCC	AUC
1	93.92	97.23	99.74	0.97	0.99
2	96.11	97.97	99.90	0.98	1.00
3	93.87	97.00	99.12	0.98	0.99
4	94.68	97.26	99.32	0.98	0.99
5	95.03	97.58	97.22	0.98	0.99
6	96.26	98.72	97.59	0.98	1.00
7	93.38	99.00	98.32	0.98	0.98
8	94.04	97.00	97.23	0.97	0.99
9	96.24	98.30	97.57	0.99	1.00
10	93.34	96.00	96.97	0.99	0.98
11	94.94	97.32	96.63	0.97	0.99
12	93.41	99.80	99.01	0.98	0.99
13	93.72	99.00	99.91	0.98	0.99
14	93.90	95.11	99.89	0.98	0.99
15	94.09	96.44	99.23	0.98	0.99
16	96.12	98.00	99.12	0.98	1.00
17	94.25	96.79	98.90	0.98	0.99
18	95.15	97.70	97.26	0.98	0.99
19	94.17	96.57	99.32	0.98	0.99
20	95.60	97.82	97.34	0.97	1.00
Average	94.61	97.80	99.89	0.98	1.00

**Table 4:** Results for independent dataset testing of three different methods.

Splits	Precision metrics				
	Acc (%)	Sp (%)	Sn (%)	MCC	AUC
90/10	95.27	94.50	96.90	0.8990	0.92
80/20	91.57	92.60	93.40	0.8310	0.89
70/30	88.10	91.34	92.10	0.8120	0.86
Average	91.65	92.81	94.13	0.8473	0.89

**Table 5:** Comparative performance of *β*Lact-Pred as compared to the previous predictors.

Predictor/identifier	Total number of *β*-lactamases	Predicted *β*-lactamases
*β*Lact-Pred	75	62
BlaPred [12]	75	58
PredLactamase [32]	75	40

**Table 6:** Comparative analysis of 10-fold cross-validation results with CNN-BLPred.

Predictor/identifier	Precision metrics			
	AUC	Sp (%)	Sn (%)	MCC
CNN-BLPred [33]	1.00	95.73	99.90	0.96
*β*Lact-Pred	1.00	97.80	99.89	0.98

## Data Availability

The data used in this paper are available from the corresponding author upon request.
